# Co-mutations of TP53 and KRAS serve as potential biomarkers for immune checkpoint blockade in squamous-cell non-small cell lung cancer: a case report

**DOI:** 10.1186/s12920-019-0592-6

**Published:** 2019-10-16

**Authors:** Cheng Fang, Chu Zhang, Wei-Qing Zhao, Wen-Wei Hu, Jun Wu, Mei Ji

**Affiliations:** Departments of Oncology, the Third Affiliated Hospital of Soochow University, Changzhou, 213003 China

**Keywords:** Squamous-cell non-small cell lung cancer, Immunotherapy, TP53, KRAS

## Abstract

**Background:**

Unprecedented durable responses are identified in clinical studies to target the signaling of programmed cell death protein-1 (PD-1) as well as its ligand (PD-L1) in patients with squamous-cell non-small cell lung cancer (NSCLC). However, factors predicting the patient subtypes that are responsive to PD-1/PD-L1inhibitors have not been fully understood yet. Biomarkers, like PD-L1 expression, tumor mutational burden(TMB), DNA mismatch repair deficiency (dMMR), and tumor-infiltrating lymphocytes (TILs), have been utilized to select patients responsive to PD-1/PD-L1inhibitors in the clinic, but each of them has limited use. Lung adenocarcinoma patients with a mutation of TP53 or KRAS, particularly those with co-mutations of TP53 and KRAS, can benefit from anti–PD-1 treatment.

**Case presentation:**

In this study, the co-mutations of TP53 and KRAS in a 64-year-old non-smoking man with squamous-cell NSCLC patient was described using the next-generation sequencing (NGS) technology. The patient was treated with the pembrolizumab combined with gemcitabine as the salvage therapy, and a marked partial response could be attained, which had persisted for over 7 months.

**Conclusion:**

In addition to testing common driving genes, like EGFR, ALK, ROS1 and BRAF, both TP53, and KRAS should also be considered in advanced or metastatic squamous-cell NSCLC.TP53 and KRAS co-mutations in squamous-cell NSCLC can be a potential factor to assess possible response to PD-1 blockade immunotherapy, but further studies with more cases are needed to confirm the prediction power.

## Background

Tremendous durable responses have been recognized in recent clinical trials to anti-programmed cell death 1 (PD-1), PD-1 ligand (PD-L1), and anti-cytotoxic T-lymphocyte antigen 4 (CTLA-4) antibody, which is used as the single agents or are combined with standard chemotherapy to treat patients who have never received treatment or those that are treated due to the advanced squamous-cell non-small cell lung cancer (NSCLC) previously [1, 2]. Nonetheless, not all patients can benefit from immunotherapy. Meanwhile, 13.8% of NSCLC patients at the advanced stage receiving PD-1/PD-L1 inhibitors treatment are reported to sustain accelerated tumor growth during immunotherapy, which is defined as the hyperprogressive disease (HPD) [3]. Therefore, it is urgent to identify biomarkers for determining the response to PD-1/PD-L1 inhibitors treatment. Typically, biomarkers, including PD-L1 expression [4], tumor mutational burden (TMB) [5], DNA mismatch repair (MMR) deficiency [6], and tumor-infiltrating lymphocytes (TILs) [7], have been used to select patients suitable for PD-1 blockade immunotherapy in clinic, but all of them have limited utility. In addition, the mutations of TP53 and KRAS in lung adenocarcinoma can be used as the underlying predictors for instructing the PD-1 blockade immunotherapy [8]. In this study, one squamous-cell NSCLC case with co-occurring TP53 and KRAS mutations was reported, who had shown a durable response to the PD-1 antibody combined with chemotherapy.

## Case presentation

**Fig. 1:**
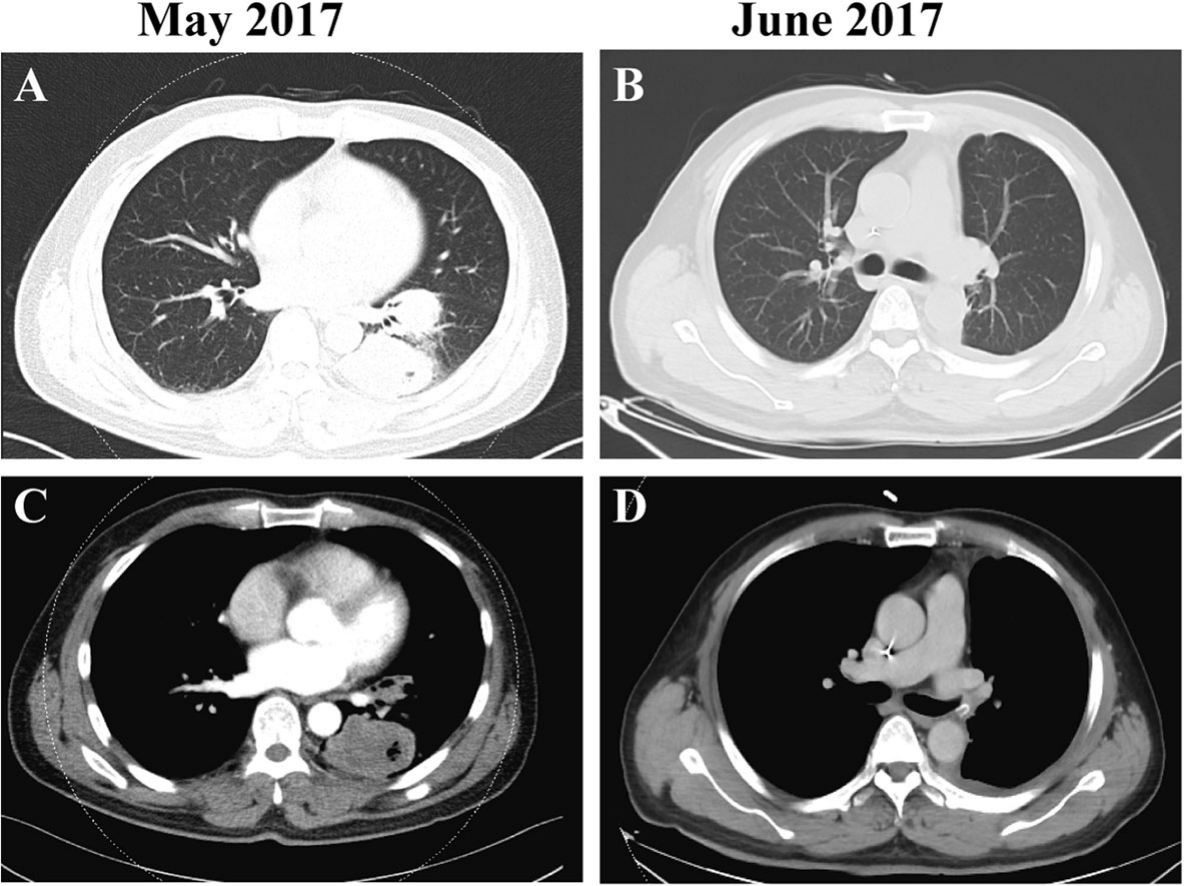
CT scans of the patient. **a**, **c** CT scan before lobectomy. Two masses in the left lower lung lobe were noted; and **b**, **d** CT scan after lobectomy

**Fig. 2:**
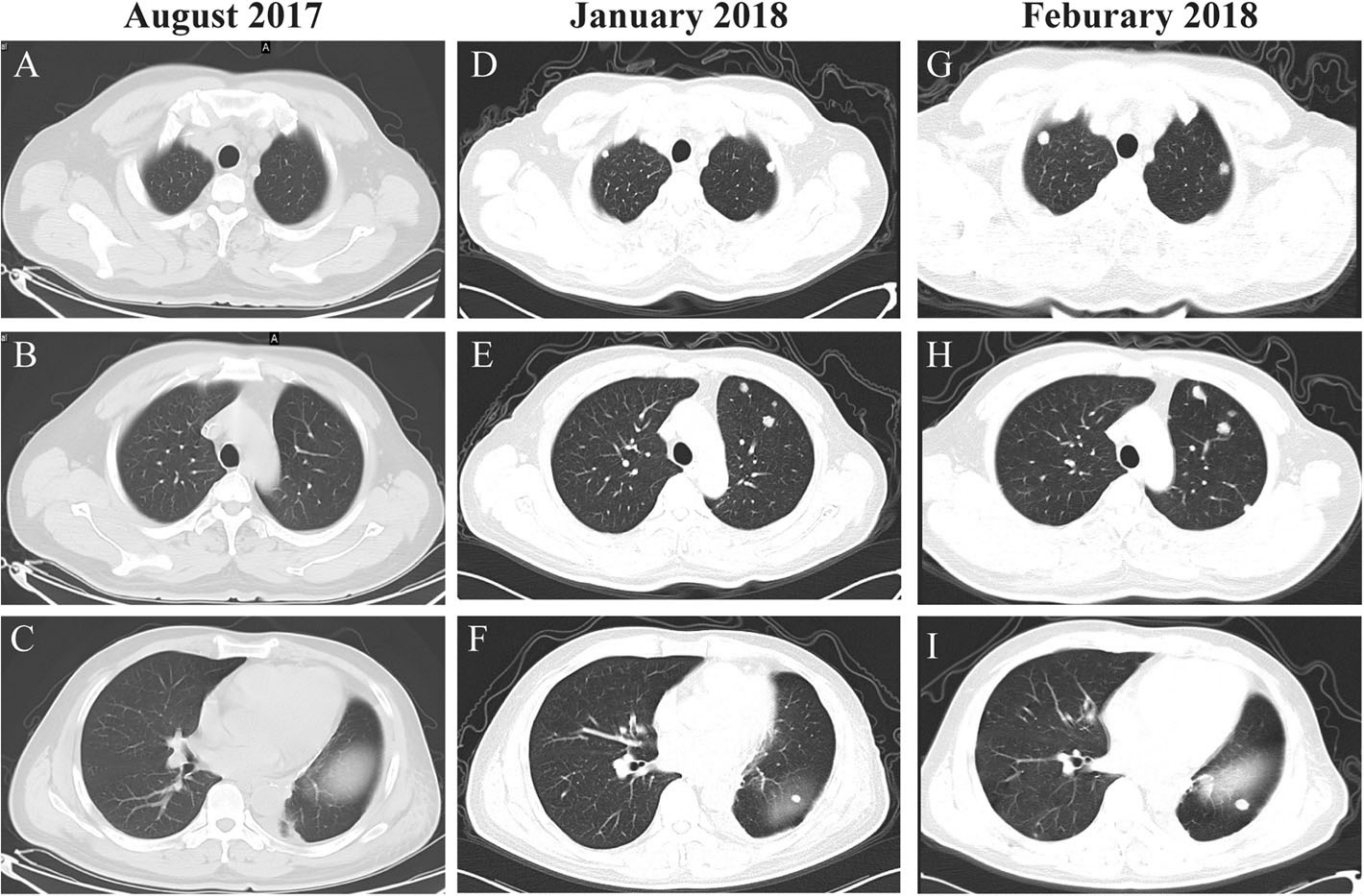
CT scans of the patient. **a**, **b**, **c** CT scan after four cycles of adjuvant chemotherapy with paclitaxel and carboplatin in August 2017. None metastases were observed in the lung or mediastinal lymph node. **d**, **e**, **f** CT scan 5 months after adjuvant chemotherapy, from which many new lung metastases in the entire lung were noted. **g**, **h**, **i** CT scan after two cycles of rh-endostain and docetaxel chemotherapy. The lung metastases became evidently larger

**Fig. 3:**
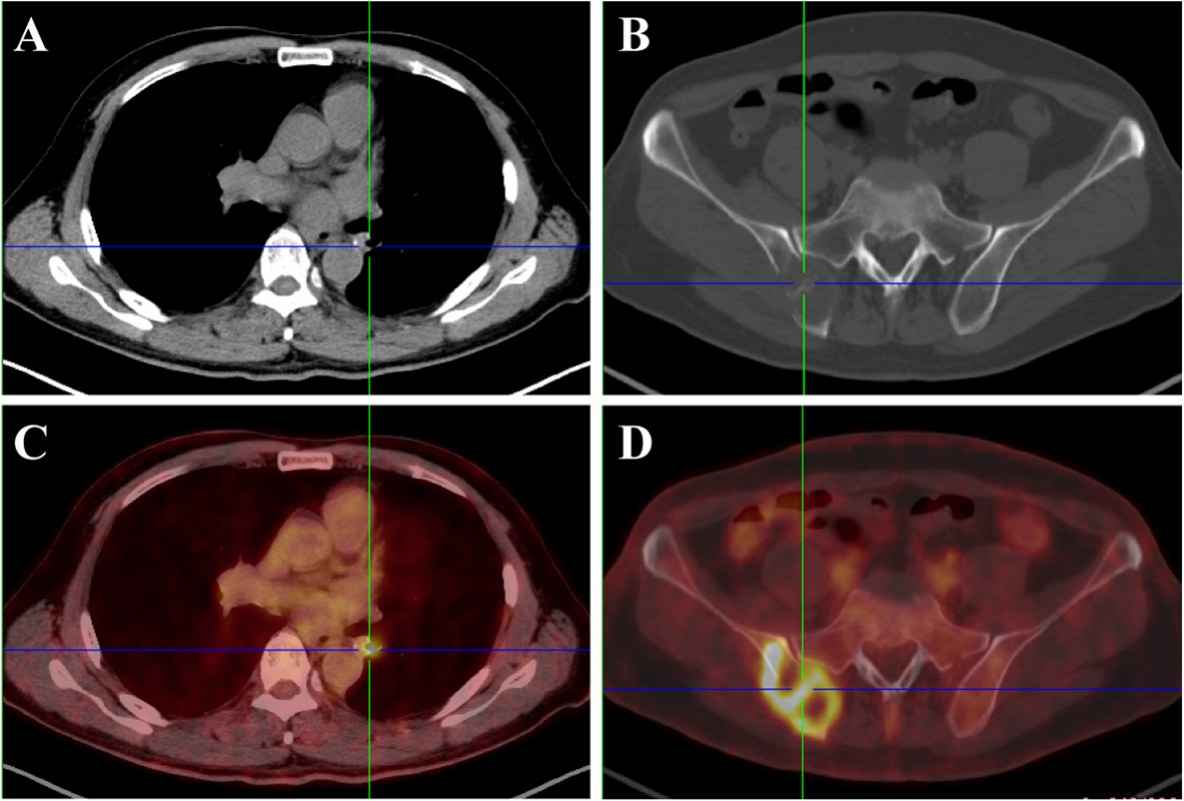
PET/CT scan showed typical imaging alternations in the anastomotic region (**a**, **c**) and right iliac bone region (**b**, **d**), which was suspected of disease recurrence

**Fig. 4:**
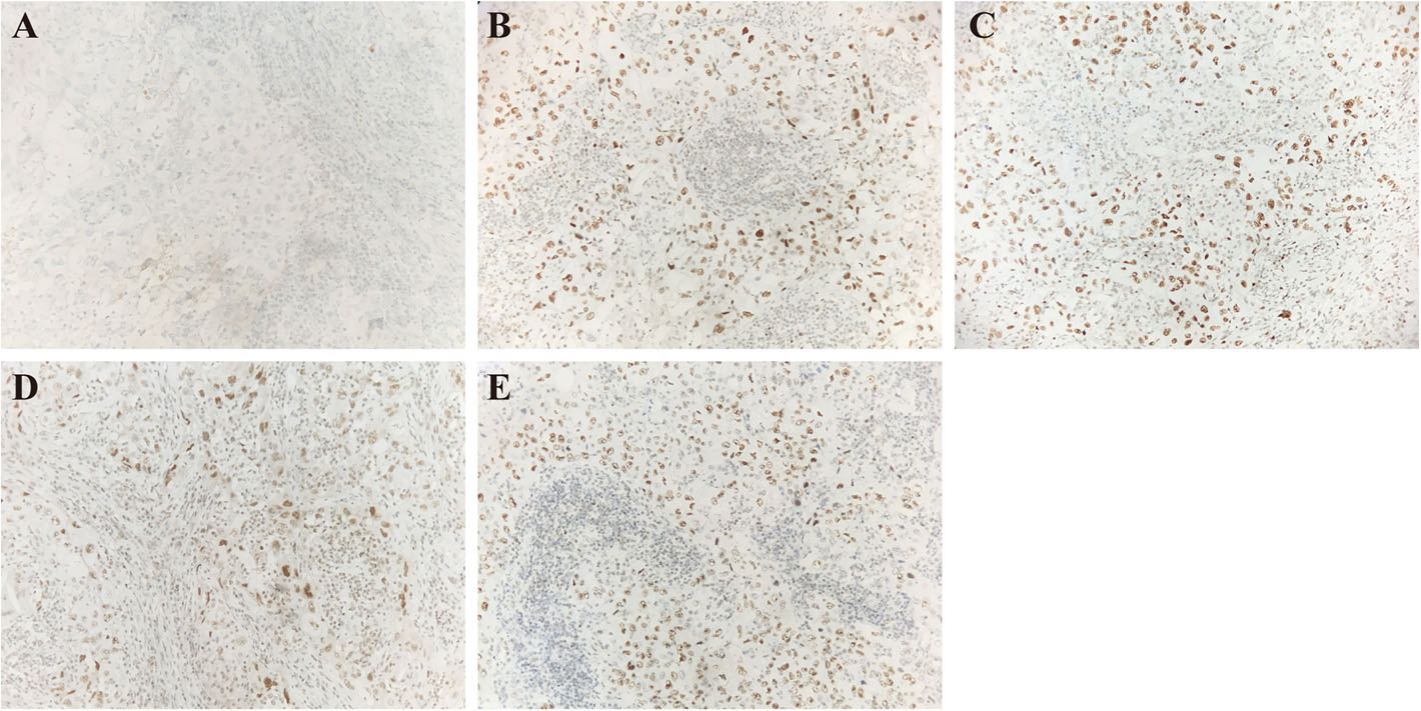
Pathological examination of postoperative tumor tissue revealed of lung adenocarcinoma and immunostaining of PD-L1 (**a**) and MMR-related proteins, including MSH2 (**b**), MSH6 (**c**), MLH1 (**d**), and PMS2 (**e**) in postoperative tumor tissue

**Fig. 5:**
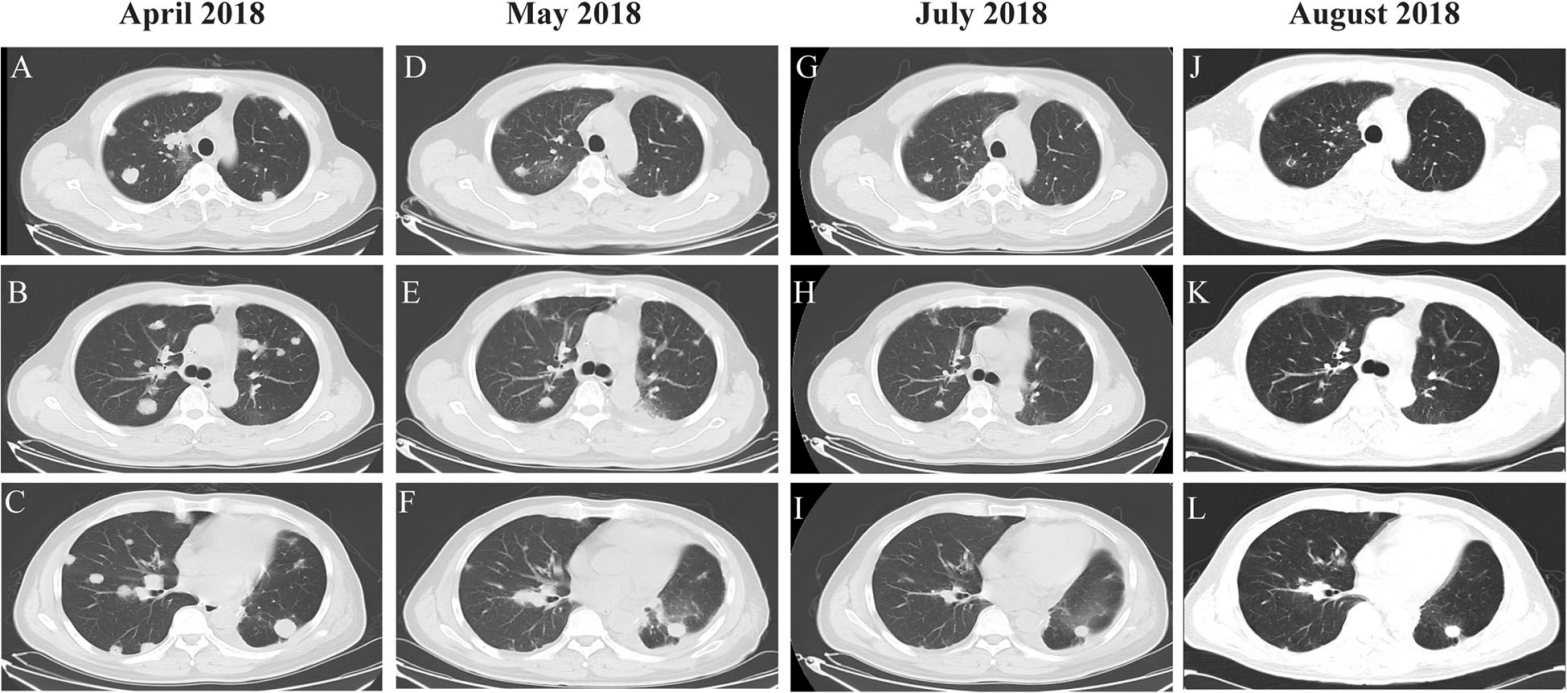
CT revealed a decreased size and number of lung nodules. Lung metastases in the entire lung were noted (**a**, **b**, **c**); CT scan after two cycles (**d**, **e**, **f**), four cycles (**g**, **h**, **i**) and six cycles (**j**, **k**, **l**) of pembrolizumab plus gemcitabine, the masses were markedly smaller and a marked response lasting for over 7 months

A 64-year-old non-smoking man was admitted into our hospital as a result of a mass in the left lower lung lobe identified by chest radiography in physical examination. Chest CT revealed only two masses in the left lower lung lobe, with no metastases in the mediastinal lymph nodes (Fig.1a, c). The patient was thus performed lobectomy of the left lower lung. Mediastinal lymph node dissection (LND) demonstrated no evidence of residual lung squamous-cells (Fig.1b, d), but 2 of 4 interlobar lymph nodes were involved, and the lesions were staged as pT3N1M0, IIIA squamous-cell NSCLC. Afterward, the patient had received adjuvant chemotherapy using paclitaxel and carboplatin for four cycles from June 2017 to August 2017. Chest CT in August 2017 revealed no metastases in the lung or mediastinal lymph node (Fig. 2a, b, c). Three months after treatment, the CEA level was found to increase to 61.61 ng/ml. In addition, positron emission tomography-CT revealed fludeoxyglucose F^18^-positive lesions in the anastomotic region (Fig.3a, c) and right iliac bone region (Fig. 3b, d), respectively, which were suspected of disease recurrence. Moreover, tests for EGFR mutation and ALK fluorescence in situ hybridization showed negative results. To alleviate bone pain, palliative radiotherapy was applied in both the anastomotic region and the right iliac bone region. However, chest CT in January 2018 revealed many new lung nodules in the entire lung (Fig. 2d, e, f). The patient was then treated with rh-endostain (30 mg d1–7 CIV) combined with docetaxel (120 mg d4 IV) every 3 weeks. Eventually, the patient suffered from rapid disease progression and symptom aggravation, cough, and dyspnea were included (Fig. 2g, h, i). Biomarkers, including PD-L1 expression, tumor TMB, and high microsatellite instability, could be used to forecast the therapeutic effect of immune checkpoint blockade [9]. To further identify the potential therapeutic targets, immunohistochemistry was used to analyze the PD-L1 and MMR-related proteins, including MSH2, MSH6, MLH1, and PMS2 (Fig. 4). PD-L1 expression was determined using the companion diagnostic PD-L1 IHC 22C3 pharmDx assay. PD-L1 expression was determined based on percentage of tumor cells with positive membranous staining and was reported as the tumor proportion score (TPS). Among them, PD-L1 was weakly stained, TPS was 2%; by contrast, four MMR-related proteins were strongly stained, suggesting no MMR deficiency in the tumor. Moreover, the next-generation sequencing (NGS) technology, along with a gene panel involving 416 genes associated with cancer, was utilized to analyze the postoperative tumor samples from patients in January 2018. The large panel can comprehensively and accurately detect variations involving gene mutations, amplifications, and fusions with clear clinical relevance to tumors. Typically, the libraries were prepared using the Hyper Prep Kit (Kapa) before sequencing on the Hiseq 4000 NGS platforms (Illumina). More importantly, the co-mutations of TP53 (pY220C, MAF = 11.07%) and KRAS (pG12V, MAF = 19.37%) were detected in the tumor; Unfortunately, the results showed no detected mutations in core genes associated with lung cancer (EGFR, ALK, c-Met, ROS1, BRAF and STK11). TMB was referred to as the total counts of non-silencing somatic mutations in the coding regions. Panel TMB was counted by summing all base substitutions and indels in the coding region of targeted genes, including synonymous alterations to reduce sampling noise and excluding known driver mutations as they are over-represented in the panel, as previously described [10].In our case, the TMB was 3.2 mutations/Mb (< 10), which belonged to the low tumor mutational burden (TMB-low). Therefore, a decision was made to proceed with chemotherapy combined with immunotherapy according to the following regimen, gemcitabine (1.4 g d1,8 IV) combined with pembrolizumab (150 mg d1 IV) every 3 weeks for 4 cycles, before pembrolizumab (150 mg) every 3 weeks since April 2018. The symptoms of cough and dyspnea in the patient were reported to be substantially alleviated. After two cycles of treatments, CT revealed a decreased size and number of lung nodules, and the response had persisted at 7 months, which had met the criteria of RECIST partial response (Fig.5).

## Discussion and conclusions

For squamous-cell NSCLC patients at the advanced stage that develop disease progression in or after first-line chemotherapy, there are limited treatment options. PD-1 inhibitors have demonstrated more remarkable survival benefit than chemotherapy in patients who are previously treated for their advanced squamous-cell NSCLC, with the 3-year survival of 17% [11]. Typically, the rationale for combined immunotherapy with chemotherapy depends on the hypothesis that cytotoxic chemotherapy will indiscriminately kill normal and cancer cells, while immunotherapy can “rev up” the immune system against cancer cells. In this study, one case with squamous-cell NSCLC was reported; He had developed co-occurring TP53 and KRAS mutations and was treated by pembrolizumab-combined gemcitabine. Fortunately, the patient had developed substantial responses to the treatment, which could last for over 7 months (until the time of submitting this manuscript).

Biomarkers, such as PD-L1 expression, TMB, MMR deficiency, and CD8^+^ T-cell infiltrate intensity, are recently proposed to be the predictors of the response to PD-1 blockade immunotherapy [9]. This patient received chemotherapy after relapse, and all markers related to the PD-1 blockade immunotherapy, such as weak PD-L1 expression, MSI and TMB-low, had indicated invalid immunotherapy. However, co-mutations of TP53 (pY220C, MAF = 11.07%) and KRAS (pG12V, MAF = 19.37%) were detected in the tumor. Dong et al. [8] reported that co-mutations of TP53 and KRAS had exerted evident effects on up-regulating PD-L1 expression, accelerating T-cell infiltration and enhancing tumor immunogenicity. More importantly, lung adenocarcinoma patients with TP53 or KRAS mutation, especially those with co-mutations of TP53 and KRAS, can favorably benefit from the anti–PD-1 treatment. In our case, the patient was resistant to docetaxel, so he was treated with pembrolizumab combined with gemcitabine, achieved significant tumor shrinkage. It seems neither one is the answer since our patient (squamous-cell NSCLC) has weak PD-L1 expression in the tumor and low TMB of 3.2 mutations/Mb. It may be the first case of TMB-L/PD-L1 low expression and TP53 and KRAS mutant lung squamous cell NSCLC patients have a long-term benefit of immunotherapy. Co-mutations of TP53 and KRAS may also be used as the promising biomarkers in immune checkpoint blockade for squamous-cell NSCLC. The patient with co-mutations of TP53 and KRAS had low PD-L1 expression, but the tumors shrank significantly, and the underlying mechanism should be further studied. In addition, there are still many treatment responses apart from the factors mentioned above; therefore, more effective biomarkers are still needed for the PD-1 blockade.

As shown in two phases III trials, for advanced squamous-cell NSCLC patients, the progression-free survival (PFS) and overall survival (OS) were improved, and using PD-1 inhibitors in combination with chemotherapy had displayed favorable safety over chemotherapy [1, 12]. Moreover, a recent report from the three-phase IMpower131 trial suggested that squamous-cell NSCLC patients at the advanced stage could benefit more from the first-line treatment combined with atezolizumab plus chemotherapy than chemotherapy alone. At the landmark of 12-month PFS, the PFS in the group receiving atezolizumab combined with chemotherapy was doubled compared with that in the group undergoing chemotherapy alone (24.7% vs. 12.0%, respectively). Such a benefit could be seen in all patients regardless of the PD-L1 expression status. However, the difference was most significant in the high PD-L1 expression group (48 and 20%, respectively). Typically, it was one of the first phase III trials that suggested that the PFS was markedly improved in squamous-cell NSCLC patients at the advanced stage treated in combination with immunotherapy [12]. Besides, KEYNOTE-407 was presented, showing that pembrolizumab combined with carboplatin and paclitaxel or nanoparticle albumin-bound (nab)-paclitaxel could improve both OS and PFS in metastatic squamous-cell NSCLC patients, compared with those receiving chemotherapy alone, irrespective of the PD-L1 expression status. In addition, the median OS was 15.9 and 11.3 months in pembrolizumab and placebo groups, respectively. The OS benefit was consistent regardless of the PD-L1 expression levels, with the hazard ratios of 0.61 among patients with the PD-L1 expression of < 1% and 0.65 among those with the PD-L1 expression of ≥1% [1].

Taken together, PD-L1 expression should be assessed in the tissues of all squamous-cell NSCLC patients at the advanced stage, and for patients with the PD-L1 expression of 50% or higher in the tumor, pembrolizumab can be used as the first-line therapy; whereas treatment for those with the PD-L1 expression of less than 50% should be started with pembrolizumab combined with carboplatin and paclitaxel. Besides, to test common driving genes, like EGFR, ALK, ROS1, and BRAF, both P53 and KRAS should also be considered. Nonetheless, the underlying mechanism of the favorable effect of immunotherapy for squamous-cell NSCLC patients with TP53 and KRAS co-mutations should be further studied. Additionally, further studies of first-line and later-line immunotherapy with chemotherapy should be carried out in larger populations.

## Data Availability

The datasets used and/or analyzed during the current study are available from the corresponding author on reasonable request.

## Abbreviations

CTLA-4: Cytotoxic T-lymphocyte antigen 4
dMMR: Mismatch repair deficiency
HPD: Hyperprogressive disease
LND: Lymph node dissection
NGS: Next-generation sequencing
OS: Overall survival
PD-1: Programmed cell death protein-1
PD-L1: Programmed cell death protein ligand − 1
PFS: Progression-free survival
TILs: Tumor-infiltrating lymphocytes
TMB: Tumor mutational burden
